# No Evidence of Persistence or Inheritance of Mitochondrial DNA Copy Number in Holocaust Survivors and Their Descendants

**DOI:** 10.3389/fgene.2020.00087

**Published:** 2020-03-03

**Authors:** Na Cai, Monika Fňašková, Klára Konečná, Miloslava Fojtová, Jiří Fajkus, Eve Coomber, Stephen Watt, Nicole Soranzo, Marek Preiss, Ivan Rektor

**Affiliations:** ^1^ Wellcome Sanger Institute, Wellcome Genome Campus, Hinxton, United Kingdom; ^2^ European Bioinformatics Institute (EMBL-EBI), Wellcome Genome Campus, Hinxton, United Kingdom; ^3^ Neuroscience Centre, CEITEC, Masaryk University, Brno, Czechia; ^4^ 1st Neurology Department, Hospital St Anne and School of Medicine, Masaryk University, Brno, Czechia; ^5^ Mendel Centre for Plant Genomics and Proteomics, CEITEC, Masaryk University, Brno, Czechia; ^6^ National Centre for Biomolecular Research, Faculty of Science, Masaryk University, Brno, Czechia

**Keywords:** mitochondrial DNA, posttraumatic stress disorder, copy number variation, quantitative PCR, Holocaust-psychic trauma

## Abstract

Mitochondrial DNA copy number has been previously shown to be elevated with severe and chronic stress, as well as stress-related pathology like Major Depressive Disorder (MDD) and post-traumatic stress disorder (PTSD). While experimental data point to likely recovery of mtDNA copy number changes after the stressful event, time needed for full recovery and whether it can be achieved are still unknown. Further, while it has been shown that stress-related mtDNA elevation affects multiple tissues, its specific consequences for oogenesis and maternal inheritance of mtDNA has never been explored. In this study, we used qPCR to quantify mtDNA copy number in 15 Holocaust survivors and 102 of their second- and third-generation descendants from the Czech Republic, many of whom suffer from PTSD, and compared them to controls in the respective generations. We found no significant difference in mtDNA copy number in the Holocaust survivors compared to controls, whether they have PTSD or not, and no significant elevation in descendants of female Holocaust survivors as compared to descendants of male survivors or controls. Our results showed no evidence of persistence or inheritance of mtDNA changes in Holocaust survivors, though that does not rule out effects in other tissues or mitigating mechanism for such changes.

## Introduction

Mitochondrial DNA (mtDNA) occurs in hundreds to thousands of copies in each cell. The levels of mtDNA is tissue-specific (Robin and Wong, 1988; Falkenberg et al., 2007; Kelly et al., 2012), and dependent on genetic factors (Scarpulla, 2008; Cai et al., 2015a; Kukat et al., 2015) and environmental stimuli. Increase in mtDNA copy number has been found to be associated with a wide range of psychological stress, including childhood parental loss, maltreatment (Tyrka et al., 2016; Ridout et al., 2019), sexual abuse (Cai et al., 2015b) and a wide range of stressful events over one’s lifetime (Cai et al., 2015b). As such, alterations to mitochondrial function are increasingly investigated as a key mechanism underlying stress-related conditions (Zhang et al., 2006; Manoli et al., 2007; Su et al., 2008). An increase in mtDNA levels obtained from blood samples has been reported in recurrent Major Depressive Disorder (MDD) (Cai et al., 2015b; Edwards et al., 2016) and a decreased in mtDNA levels has been shown in moderately severe post-traumatic stress disorder (PTSD) (Bersani et al., 2016). Other reports showing conflicting results. The former has been shown to be more pronounced in those experiencing an episode of MDD than those with a history of it, and are reversible upon cessation of the stressful stimuli in animal models (Cai et al., 2015b), demonstrating both disease state-dependence and potential for recovery. Among individuals with a history of severe recurrent MDD, chronicity of disease state was positively associated with mtDNA levels (Edwards et al., 2016), suggesting complete recovery may not be achieved or requires a long time.

Stress-related changes in mtDNA may be mediated by an alteration of hypothalamic–pituitary–adrenal (HPA) axis reactivity (Cai et al., 2015b), likely partly accounting for mtDNA changes seen in both MDD (Holsboer, 2000; Kloet et al., 2005) and PTSD (van Zuiden et al., 2012). Other biological processes have been proposed as mechanisms for stress-related changes in mtDNA, including mitochondrial biogenesis mediated by stress-induced increase in reactive oxygen species (ROS) (St-Pierre et al., 2006; Scarpulla et al., 2012), and mtDNA damage induced apoptosis and release of mtDNA (Lindqvist et al., 2018). A range of broad, converging or co-existing pathways may explain inconsistencies between studies primarily capturing effects of different pathways (Kageyama et al., 2018; Tymofiyeva et al., 2018; Verhoeven et al., 2018; Wang et al., 2018; Tsujii et al., 2019; Czarny et al., 2019), an increasing number of conditions associated with changes in mtDNA levels (Carew et al., 2004; Lan et al., 2008; Malik et al., 2009), and tissue-specificity of mtDNA changes (Cai et al., 2015b).

The mechanism of intergenerational transmission of trauma is not understood. One possibility is that the transmission is based on social mechanisms, another is it is mediated by genetic or epigenetic changes. While animal models have shown that stress-related mtDNA changes likely affect multiple tissues including the ovary (Cai et al., 2015b), it is unknown if stress has an effect on mtDNA in mature oocytes in females, and if such changes may be inherited. It is widely recognized that there is an mtDNA bottleneck during the development of primordial germ cells in female human embryos (Giles et al., 1980), followed by a gradual thousand-fold expansion (Cotterill et al., 2013) of mtDNA during oogenesis (Jenuth et al., 1996; Reynier et al., 2001; Barritt et al., 2002; Cree et al., 2008; Wai et al., 2008). However, little is known of whether maternal stress in the prenatal or gestational period would affect the mtDNA bottleneck or mtDNA expansions during oogenesis. Though placental mtDNA levels at birth has been found to be inversely correlated with maternal prenatal negative events, PTSD, depressive symptoms and lifetime stress (Brunst et al., 2017), it remains unclear whether maternal stress has any lasting effect on mtDNA levels in descendants and their health and fertility (Reynier et al., 2001; Santos et al., 2006).

In this study, we investigate whether mtDNA increases during a stressful life event may persist throughout one’s lifetime and affect the mtDNA levels of one’s descendants, and whether that is dependent on the development of a persistent stress-related pathology. We assess the relative levels of mtDNA obtained from peripheral blood mononuclear cells (PBMC) of Holocaust survivors in Czech Republic, most of them from the Jewish communities of Brno and Prague, and of two generations of their descendants, many of whom suffer from PTSD, with age-matched individuals from the same generations who were not exposed to this extreme circumstance and its consequences (Konečná et al., 2019). The Holocaust survivors represent a group of people who have gone through extreme physical and psychological trauma early in life, and have lived to a relatively old age. We took the unique opportunity to examine this specific group of people in this study.

## Results

### Holocaust Survivors and Their Descendants

**Figure 1:**
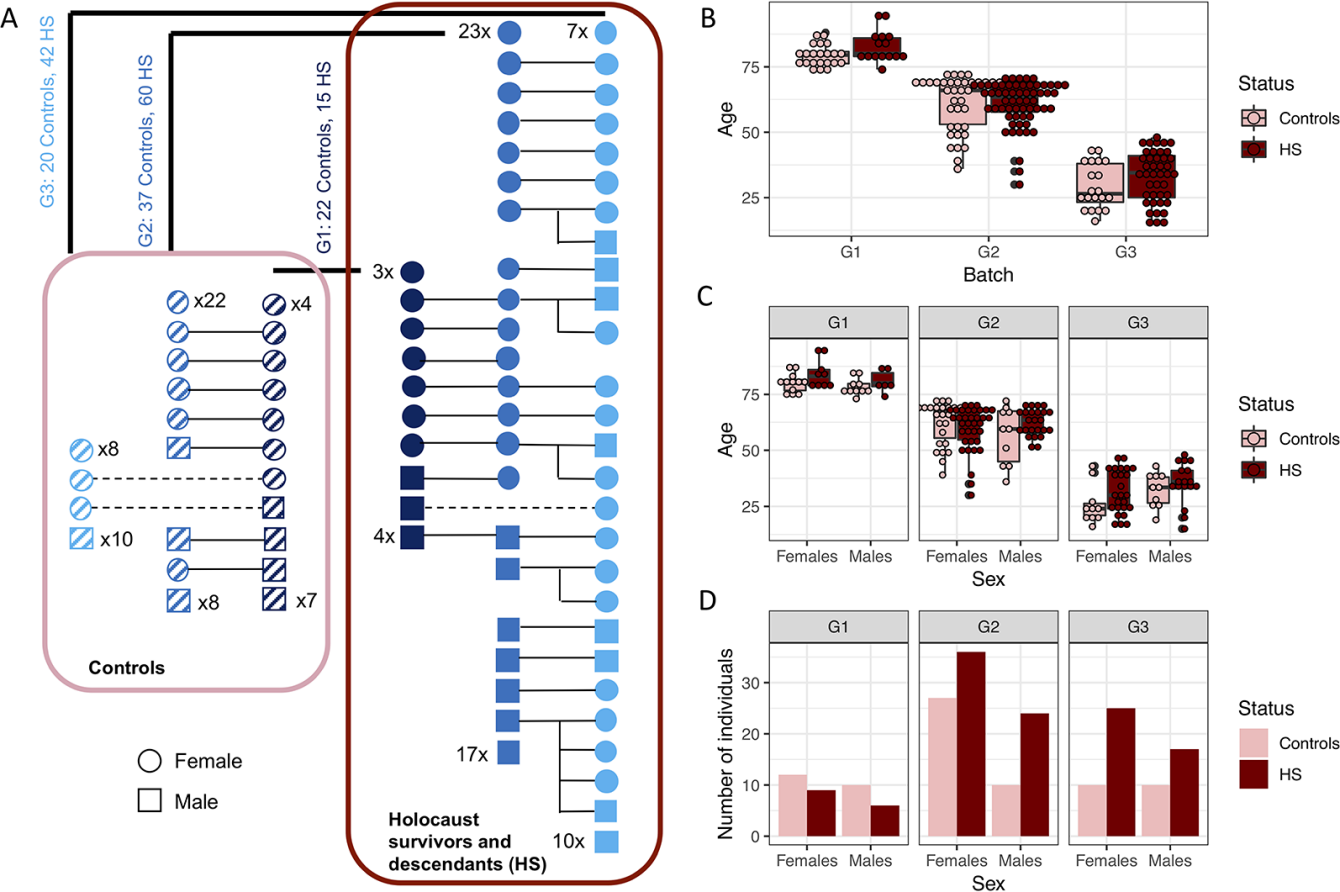
Study participants. **(A)** Schematic diagram showing relationships between generation 1 (G1) Holocaust survivors and controls with generations 2 and 3 (G2 and G3) descendants. Males are depicted as squares and females as circles; Holocaust survivors and their descendants are depicted colored solid navy, blue and cyan squares and circles for G1, G2 and G3 respectively, and controls of each generation are depicted as squares and circles filled with slanted dashes. Numbers of those individuals without first degree relationships with other individuals in this study are written for each generation. All Holocaust survivors and their descendants are encircled by a dark red rectangle, while all controls are encircled by a pink rectangle. Numbers of Holocaust survivors or their descendants and controls are written for each generation. **(B)** Boxplot of the age of Holocaust survivors and their descendants (in dark red), and controls (in pink), for each generation. **(C)** Boxplot of age of female and male Holocaust survivors and their descendants (in dark red) and controls (in pink) for each generation. **(D)** This figure shows the number of female and male Holocaust survivors and their descendants (in dark red) and controls (in pink) participating in this study with mtDNA successfully quantified through qPCR.

We obtained DNA samples from peripheral blood mononuclear cells (PBMCs, see *Methods*) from 235 individuals recruited for this study, 196 of which passed DNA quality control and were successfully analysed with quantitative polymerase chain reaction (qPCR) for mitochondrial DNA (mtDNA) copy number (*Methods*) and used for all analysis in this manuscript. Seventy seven of these individuals were men and 119 women. One hundred seventeen of them were first generation (G1) Holocaust survivors or their second (G2) and third generation (G3) descendants (n = 15, 60 and 42 for G1–3 respectively) and 79 (n = 22, 37, 20 for G1–3 respectively) were controls from all three generations (**Figure 1A**). Using this sample, we have 0.8 statistical power to find effect sizes of Holocaust survivor status on mtDNA copy number that are larger than 0.48, 0.29 and 0.32 in G1 to G3 using linear regression respectively. We will not have adequate power to detect effects smaller than this.

Holocaust survivors and their descendants are not significantly different in terms of age from the controls as a whole cohort (t-test P = 0.148, **Figure 1B**), in individual generations (t-test P = 0.048, 0.982 and 0.105 respectively for G1–3), or in each sex (t-test P = 0.095 and 0.855 in males and females respectively, **Figure 1C**). While there are different numbers of men and women in each generation, there is no significant difference by sex between the number of controls and Holocaust survivors and their descendants across generations (Fisher’s exact test P = 0.768) or within individual generations (G1 Fisher’s exact test P = 1; G2 Fisher’s exact test P = 0.27; G3 Fisher’s exact test P = 0.59, **Figure 1D**).

### mtDNA Copy Number Is Associated With Age and Sex

**Figure 2:**
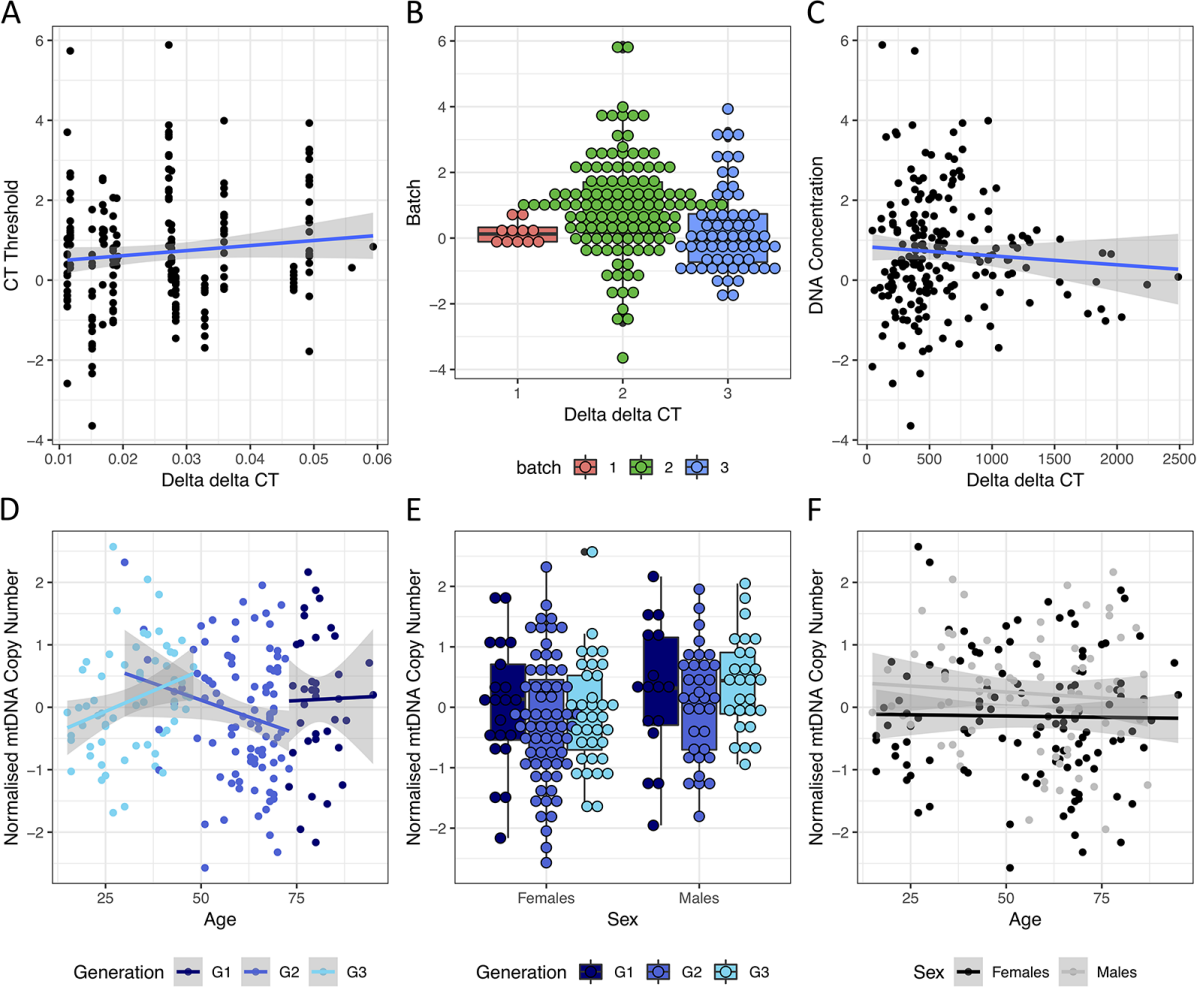
Effects of technical and biological covariates on mtDNA copy number measurements. **(A)** Relationships between ΔΔCT values, representing raw mtDNA copy number measures from qPCR, and the threshold CT values for each qPCR run. **(B)** Boxplot of ΔΔCT values from each qPCR batch. **(C)** Relationship between ΔΔCT values and starting DNA concentration (before dilution) of each sample. **(D)** Relationship between normalized mtDNA copy number measure (after correcting for threshold CT values, qPCR batch and starting DNA concentration) and age in individuals from G1, G2 and G3 in navy, blue and cyan respectively. **(E)** Boxplot of normalized mtDNA copy number in female and male individuals from G1, G3 and G3 in navy, blue and cyan respectively. **(F)** Relationship between normalized mtDNA copy number and age in females (in black) and males (in grey) respectively.

mtDNA copy number was quantified using qPCR on 196 individuals across three batches and 17 plates, each with a different threshold quantification cycle (CT threshold). As individuals were randomized across the plates, there was no significant difference between the plates on sex (Chi-squared test P value = 0.856), holocaust experience (Chi-squared test P value = 0.396), or PTSD status (Chi-squared test P value = 1). In addition to normalizing the raw mtDNA copy number measured in each plate using measures from a reference DNA sample of constant concentration (resulting in ΔΔCT values representing raw measures of mtDNA copy number, *Methods*), we further corrected the raw mtDNA copy number measure for the following: CT threshold across plates (ANOVA P value < 10^−16^, variance explained = 0.45, **Figure 2A**), PCR batch (ANOVA P value = 1.26 × 10^−4^, variance explained = 0.05, **Figure 2B**), and concentration of DNA extracted from PBMC before dilution for qPCR (P value = 0.01, variance explained = 0.01, **Figure 2C**). These capture plate effects, batch effects due to difference in reagent batches, and experimental error in dilution respectively. We then quantile normalized the residuals across all individuals to obtain our final measure of mtDNA copy number for analysis.

As both sex and age have previously been shown to contribute to variations in mtDNA copy number, we first asked if we can observe previously found trends among the controls. While mtDNA copy number is not significantly associated with age in the whole cohort (linear regression P = 0.58, beta = −0.002, se = 0.004), it significantly decreases with age in G2, the single generation with the largest age range (30–73, P = 0.05, beta = −0.02, se = 0.01, **Figure 2D**) where no individuals had personally experienced holocaust. This is consistent with previous reports demonstrating decrease of mtDNA levels with age (Mengel-From et al., 2014). We also observed significantly higher levels of mtDNA in males in the whole cohort (linear regression P = 0.01, beta = 0.37, se = 0.14, **Figure 2E**), as well as in the youngest generation G3 (age range 15–48, 35 males/27 females, linear regression P = 0.02, beta = 0.51, se = 0.21). We found no interactions between age and sex effects on mtDNA copy number in the whole cohort (interaction P value = 0.67, **Figure 2F**) or any individual generations (interaction P values = 0.985, 0.258 and 0.448 for G1–3 respectively).

### Low Variation in mtDNA Copy Number Among Haplogroups

**Figure 3:**
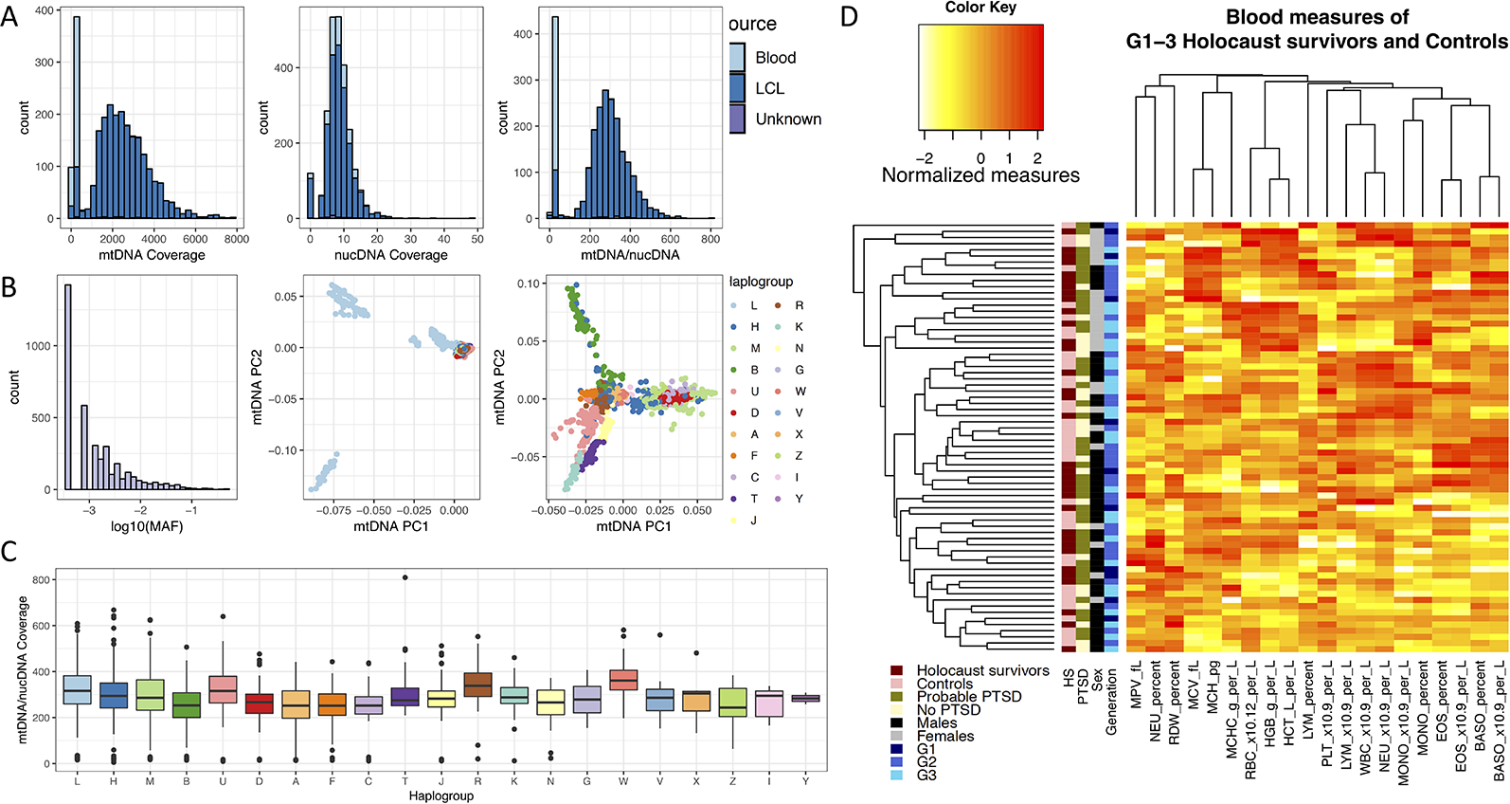
Effects of mtDNA haplogroup and blood cell count on mtDNA copy number measurements. **(A)** Left and middle panel show the sequencing coverage over mtDNA and nuclear DNA in Blood (light blue) and lymphoblastic cell lines (LCL, blue) in the 1000 Genomes Phase 3 project, and the right panel shows the ratio between them. **(B)** The left panel shows the number of SNP variants called per minor allele frequency (MAF) on the log10 scale from sequencing of mtDNA in LCL samples of 1000 Genomes Phase 3 individuals; the middle panel shows the distribution of all individuals by their first two principle components computed from mtDNA SNPs (mtDNA PC1, mtDNA PC2), colored by their mitochondrial haplogroups. Individuals of Haplogroup L (mostly of African origin) contribute the greatest mtDNA diversity as shown in the middle panel, and removing these individuals from the principle component analysis gives greater resolution for visualization of the mtDNA diversity among all other Haplogroups, as shown in the right panel. **(C)** Boxplot of effect of mtDNA Haplogroups on mtDNA copy number, estimated using the ratio of mtDNA and nuclear DNA sequencing coverage. All Haplogroups with significant effects on mtDNA copy number (L, A, B, C, D, F) are rare in European populations. **(D)** Heatmap and clustering of normalized blood cell count measures obtained from 70 individuals in this study.

As all Jewish individuals in Czech Republic were persecuted and experienced the Holocaust during World War II, one of the confounding factors in this study is the controls are not of the same origin as the Holocaust survivors, and may not have the same mtDNA Haplogroups as them. If mtDNA haplogroups have an effect on mtDNA copy number, systematic differences in mtDNA haplogroups between Holocaust survivors and controls can lead to spurious findings. We therefore investigated whether mtDNA copy number differ between Haplogroups in an independent sequencing dataset, in order to assess how likely a mismatch in mtDNA Haplogroups can lead to spurious findings. We obtained mtDNA copy numbers (*Methods*) from whole-genome sequencing of lymphoblastic cell lines (LCLs) of individuals in Phase 3 of the 1000 Genomes Project (Consortium and 1000 G. P. and The 1000 Genomes Project Consortium, 2015) (1000G) for this investigation (**Figure 3A**). To obtain the Haplogroups of each individual in 1000G, we called homoplasmic variants from the mtDNA using HaplogroupCaller in GATK v4 (*Methods*), and called Haplogroups using these variants with Haplogrep v2 (Consortium and 1000 G. P. and The 1000 Genomes Project Consortium, 2015; Weissensteiner et al., 2016) (**Figure 3B**, *Methods*).

**Figure 3C** shows the relative mtDNA copy number in different Haplogroups represented in 1000G. Testing each haplogroups against all others for association with mtDNA copy number (**Table 1**), we found that mtDNA copy number is significantly higher in Haplogroup L (beta = 0.38, se = 0.05, P = 9.43 × 10^−15^), and lower in Haplogroups A (beta = −0.50, se = 0.10, P = 3.14 × 10^−7^), B (beta = −0.54, se = 0.09, P = 6.32 × 10^−10^), C (beta = −0.53, se = 0.15, P = 3.16 × 10^-4^), D (beta = −0.38, se = 0.0.10, P = 9.31 × 10^−5^), and F (beta = −0.49, se = 0.12, P = 2.11 × 10^−5^), none of which occur at high frequencies in Europe (Torroni et al., 2000; Simoni et al., 2000). As such, it is unlikely that Haplogroup differences between Holocaust survivors and their descendants and controls, if any, would lead to spurious associations with mtDNA copy number.

**Table 1:** Effect of mtDNA Haplogroups on mtDNA copy number.

Haplogroup	Beta	SE	P value
W	0.735	0.242	2.46e−03
R	0.444	0.159	5.25e−03
L	0.381	0.049	9.43e−15^*^
U	0.263	0.089	3.29e−03
T	0.110	0.152	4.70e−01
H	0.072	0.056	2.03e−01
M	0.001	0.080	9.87e−01
K	−0.064	0.187	7.32e−01
J	−0.104	0.156	5.03e−01
V	−0.104	0.277	7.07e−01
Y	−0.112	0.705	8.74e−01
X	−0.144	0.333	6.65e−01
G	−0.194	0.243	4.24e−01
I	−0.304	0.408	4.55e−01
N	−0.368	0.224	1.01e−01
Z	−0.368	0.377	3.29e−01
D	−0.375	0.096	9.31e−05^*^
F	−0.495	0.116	2.11e−05^*^
A	−0.505	0.098	3.14e−07^*^
C	−0.535	0.148	3.16e−04^*^
B	−0.549	0.088	6.33e−10^*^

This table shows the effect (Beta) of each mtDNA Haplogroup in 1000 Genomes Phase 3 on mtDNA copy number quantified through whole-genome sequencing, ordered by their effects. The ones with significant effect after multiple testing correction for 21 Haplogroups (P value threshold = 0.0024) on mtDNA copy number are marked with asterisk (*).

### Platelet Levels in Whole Blood Sample Affect mtDNA Copy Number in Extracted PBMC

A subset of 70 individuals had their whole blood counts assessed on their blood sample prior to extraction of PBMCs and DNA (*Methods*). While there is variation among individuals in all blood measures, we found no significant effect of Holocaust, probable PTSD diagnosis, or age on individual quantile-normalized blood cell counts after multiple-testing correction (P threshold = 0.05/80 = 2.63 × 10^−4^). We did find significant effects of sex (being male) on red blood cell count (RBC, P = 1.47 × 10^−4^, beta = 0.86, se = 0.21), haemoglobin levels (HGB, P = 5.57 × 10^−7^, beta = 1.09, se = 0.20), haematocrit levels (HCT, P = 6.16 × 10^−7^, beta = 1.07, se = 0.19) and monocyte percentage (MONO_Percent, P = 2.32 × 10^−6^, beta = 0.95, se = 0.21). All results are summarized in **Table 2**. We performed hierarchical clustering on all independent quantile-normalized blood measures and found no clustering by experience of Holocaust, probable PTSD diagnosis, sex and generation (**Figure 3D**).

**Table 2:** Effect of covariates on blood cell counts.

Blood cell count	Age			Sex			Holocaust survivor status			Probable PTSD		
	Beta	SE	P value	Beta	SE	P value	Beta	SE	P value	Beta	SE	P value
WBC_x10.9_per_L	−0.001	0.006	8.98E−01	−0.302	0.234	2.01E−01	0.571	0.218	1.08E−02	0.351	0.233	1.37E−01
RBC_x10.12_per_L	−0.009	0.006	1.30E−01	**0.857**	**0.213**	**1.47E**−**04**	0.273	0.226	2.31E−01	0.424	0.231	7.11E−02
HGB_g_per_L	0.000	0.006	9.66E−01	**1.087**	**0.197**	**5.58E**−**07**	−0.008	0.228	9.71E−01	0.184	0.235	4.37E−01
HCT_L_per_L	0.002	0.006	6.90E−01	**1.067**	**0.194**	**6.17E**−**07**	−0.205	0.223	3.62E−01	0.281	0.230	2.27E−01
MCV_fL	0.018	0.005	1.18E−03	0.087	0.237	7.13E−01	−0.751	0.210	6.35E−04	−0.371	0.233	1.16E−01
PLT_x10.9_per_L	−0.001	0.006	9.13E−01	−0.820	0.215	3.02E−04	0.004	0.228	9.87E−01	−0.056	0.237	8.15E−01
MCH_pg	0.014	0.006	1.41E−02	0.228	0.235	3.35E−01	−0.506	0.220	2.45E−02	−0.473	0.230	4.33E−02
MCHC_g_per_L	−0.007	0.006	2.52E−01	0.269	0.234	2.53E−01	0.524	0.219	1.92E−02	−0.102	0.236	6.67E−01
RDW_percent	0.004	0.006	4.69E−01	−0.409	0.232	8.20E−02	0.137	0.228	5.48E−01	−0.130	0.236	5.84E-−01
MPV_fL	−0.006	0.006	2.71E−01	0.142	0.236	5.50E−01	−0.077	0.228	7.37E−01	0.128	0.236	5.90E−01
NEU_percent	0.001	0.006	8.56E−01	−0.241	0.238	3.16E−01	0.027	0.230	9.05E−01	0.194	0.237	4.15E−01
LYM_percent	−0.002	0.006	7.64E−01	−0.105	0.240	6.62E−01	0.147	0.229	5.25E−01	−0.037	0.238	8.78E−01
MONO_percent	−0.004	0.006	4.86E−01	**0.955**	**0.210**	**2.32E**−**05**	−0.339	0.226	1.39E−01	−0.351	0.234	1.39E−01
EOS_percent	0.007	0.006	2.23E−01	0.636	0.227	6.63E−03	0.028	0.230	9.04E−01	−0.435	0.232	6.45E−02
BASO_percent	0.008	0.006	1.71E−01	0.033	0.238	8.89E−01	−0.216	0.227	3.43E−01	−0.196	0.235	4.08E−01
NEU_x10.9_per_L	0.000	0.006	9.79E−01	−0.286	0.237	2.33E−01	0.441	0.224	5.30E−02	0.345	0.234	1.45E−01
LYM_x10.9_per_L	0.000	0.006	9.70E−01	−0.311	0.237	1.94E−01	0.519	0.221	2.20E−02	0.228	0.236	3.39E−01
MONO_x10.9_per_L	−0.004	0.006	4.41E−01	0.549	0.230	2.00E−02	0.227	0.228	3.23E−01	0.094	0.237	6.93E−01
EOS_x10.9_per_L	0.005	0.006	3.50E−01	0.482	0.232	4.16E−02	0.254	0.227	2.67E−01	−0.231	0.236	3.31E−01
BASO_x10.9_per_L	0.006	0.006	2.52E−01	−0.211	0.232	3.66E−01	−0.103	0.224	6.48E−01	−0.154	0.231	5.08E−01

This table shows the effect (Beta) of each covariate (age, sex, holocaust survivor status, and probable PTSD diagnosis) on each blood cell type assessed through linear regression, its standard error (SE) and its P value. The significant effects after multiple testing correction are highlighted in bold.

We tested for effects of all blood cell count measures on mtDNA copy number jointly, after controlling for age and sex. Only platelet levels showed significant effects on mtDNA copy number (PLT, P = 0.043, beta = −5.45 × 10^−3^, se = 2.61 × 10^−3^). Of note, we have specifically chosen to use DNA extracted from PBMCs instead of whole blood for this study to remove DNA contribution from platelets—platelets contain only mtDNA and no nuclear DNA, as variation in platelet levels will confound measurements of mtDNA copy number relative to nuclear DNA copy number (Hurtado-Roca et al., 2016).

### mtDNA Copy Number Does Not Index Previous Holocaust Experience or Potential PTSD Diagnosis

**Figure 4:**
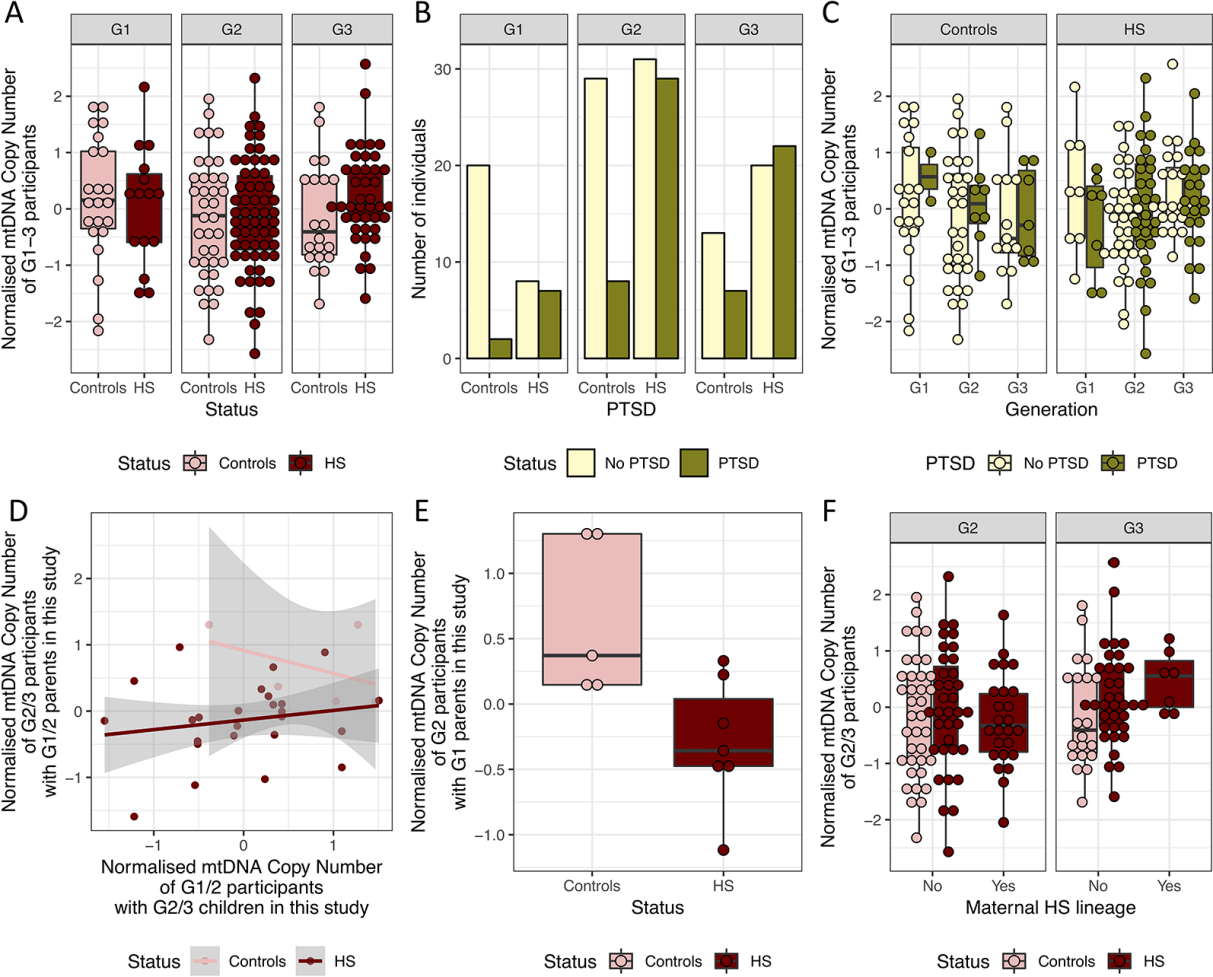
mtDNA copy number is not significantly different between HS and controls. **(A)** Boxplot of the normalized mtDNA copy number in holocaust survivors and their descendants (HS, in dark red) and controls (in pink) in G1–3. **(B)** Number of HS and controls with and without potential PTSD diagnosis from PCL-C in G1–3. **(C)** Boxplot of normalized mtDNA copy number in HS and controls who have a probable PTSD diagnosis and those who do not in each generation. **(D)** Relationship between normalized mtDNA copy number in G1/2 parents and that in their G2/3 children who are also participants in the study. **(E)** Boxplot of normalized mtDNA copy number in G2 HS and controls who are sons or daughters of G1 HS and controls in this study. Controls have significantly higher normalized mtDNA copy number. **(F)** Boxplot of normalized mtDNA copy number in all G2 and G3 HS and controls with and without maternal HS lineage, including those who are not children or grandchildren of G1 and G2 HS and controls.

Having controlled for technical and biological confounding factors, we asked if mtDNA copy number was significantly different between controls and Holocaust survivors and their descendants. We first examined the generation who has personally experienced Holocaust. In G1, we do not have adequate sample size to detect significantly higher mtDNA copy number (beta > 0.48) in Holocaust survivors than controls, after controlling for age and sex as covariates in a linear regression model at our sample size (*Methods*, P = 0.60, beta = 0.07, se = 0.14, **Figure 4A**). The same is true if the analysis was conducted separately among males and females, controlling for age as a covariate (females: P = 0.48, beta = 0.13, se = 0.19; males: P = 0.97, beta = −0.009, se = 0.22). While this is inconsistent with our hypothesis that there will be an increase in mtDNA copy number due to chronic stress during the Holocaust in Holocaust survivors, it is consistent with previous reports of the dynamic nature of mtDNA copy number increase in response to chronic stress and their reversing to normal levels with time.

As it was previously shown that persistent mtDNA copy number increase was dependent on the depressive state (Cai et al., 2015a), we asked if this is also true for PTSD. All but 10 individuals were screened for markers of PTSD were evaluated using the 17-item civilian version of the PCL questionnaire (PCL-C, *Methods*), where individual items are scored 0 to 5, and total scores range from 0 to 85. A cutoff of 30 was used to indicate potential diagnosis of PTSD. Among the 15 G1 Holocaust survivors, seven has a potential diagnosis of PTSD. We found no significant differences in mtDNA copy number in Holocaust survivors with potential diagnosis of PTSD when compared to those without a potential diagnosis of PTSD after correcting for age and sex (P = 0.67, beta = −0.08, se = 0.17) or controls (P = 0.80, beta = −0.07, se = 0.26).

We found significantly more Holocaust survivors and their descendants with PTSD (Fisher’s exact test P = 8.71 × 10^−5^, OR = 3.56, 95% CI = 1.80–7.31) than controls in the whole cohort without accounting for the different generations, in both females (Fisher’s exact test P = 0.021, OR = 2.57, 95% CI = 1.09–6.38) and males (Fisher’s exact test P = 8.39 × 10^−4^, OR = 6.04, 95% CI = 1.85–23.78). Interestingly, this trend is observed in both G1 (Fisher’s exact test P = 0.017, OR = 8.18, 95% CI = 1.21–97.32) and G2 (Fisher’s exact test P = 0.010, OR = 3.35, 95% CI = 1.23–9.92), but not in G3 (Fisher’s exact test P = 0.278, OR = 2.02, 95% CI = 0.60–7.27, **Figure 4B**). We do not find significant associations between mtDNA copy number with potential diagnosis of PTSD in G1 (P = 0.54, beta = −0.30, se = 0.50), G2 (P = 0.69, beta = 0.09, se = 0.22) or G3 (P = 0.74, beta = 0.06, se = 0.19, **Figure 4C**).

### No Evidence of Inheritance of Elevated PBMC mtDNA Levels in Descendants of Holocaust Survivors

Finally, we tested if potential previous elevation of mtDNA copy number in Holocaust survivors could be inherited by their children. Eight G2 and six G3 participants are identified as children and grandchildren of G1 Holocaust survivor participants in the study, and a further 17 G3 participants are children of 12 G2 Holocaust survivor descendant participants. Similarly, six G2 and one G3 participants are identified as children and grandchildren of G1 control participants in the study. This is summarized in **Figure 1A**.

Using all known parent–child relationships in the dataset, we found that parents’ mtDNA copy number does not significantly correlate with children’s mtDNA copy number, after controlling for age and sex of both parents and children (P = 0.30, beta = 0.47, se = 0.41). This is true when performed just in Holocaust survivors and their descendants (P = 0.07, beta = 0.32, se = 0.17, **Figure 4D**), and we do not have enough data points to perform this analysis in controls. However, we found that G1 Holocaust experience does have a significant impact on their G2 descendants’ mtDNA copy number after controlling for age and sex of both parents and children (P = 0.01, beta = −1.18, se = 0.32, **Figure 4E**). In other words, we found lower mtDNA copy number in children of G1 Holocaust survivors than in children of G1 participants who did not experience Holocaust. We get a similar result performing this analysis in only female G1 participants and their G2 descendants (P = 0.06, −1.17, se = 0.44), though the effect of parental Holocaust experience is no longer significant. We are unable to perform this analysis on their G3 participants’ mtDNA copy number, as none of them are children or grandchildren of G2 and G1 controls in the study.

Using all participants, including those without records of relationships with one or more other participants in the study, we found that G2 and G3 descendants of Holocaust survivors do not differ significantly in their mtDNA copy number from controls in their generations after controlling for age and sex (G2: P = 0.97, beta = 0.007, se = 0.21, G3: P = 0.15, beta = 0.33, se = 0.23). Further, in both G2 and G3, mtDNA copy number is not significantly different in descendants of female G1 Holocaust survivors than other individuals in their generations, after controlling for age and sex in a linear regression model (G2: P = 0.45, beta = −0.18, se = 0.23, G2: P = 0.22, beta = 0.40, se = 0.33, **Figures 4F**).

## Discussion

Holocaust was one of the most horrific periods of the twentieth century. The Holocaust survivors experienced varied forms of persecution during WWII. For all of them, it was six years lasting humiliation, deprivation of basic human rights, deportation, imprisonment in the concentration or death camps or in hiding, under false identities or in mountains or combating in partisans groups. All of them were under threat to be assassinated. Those who survived this psychological and physical ordeal continued to experience trauma due to the murdering of their families, friends and community after the war.

In this study mtDNA copy number in PBMCs obtained in HS were compared to controls without Holocaust experience. While it was impossible to determine levels of mtDNA immediately following the Holocaust experience, our study assumed that they would have been increased in Holocaust survivors, consistent with previous reports of elevated mtDNA after severe and chronic stress. Under this assumption, we asked whether their mtDNA copy number remained elevated decades after their experience, and whether it could be inherited by their descendants. After accounting for variation in mtDNA copy number that may be due to technical between qPCR runs and biological differences between individuals, we investigated the mtDNA copy number differences between G1 Holocaust survivors, their G2 and G3 descendants, and controls from G1 to G3. Bearing in mind we are limited in statistical power by the small sample size of our cohort, and we would not be able to account for effect sizes smaller than beta = 0.29, we found no significant difference in mtDNA copy number or their age-related dynamic in Holocaust survivors as compared to controls in any generation. This is consistent with findings of no significance difference in telomere length and their age-related dynamics in Holocaust survivors from the same cohort (Konečná et al., 2019), as mtDNA copy number was previously shown to be negatively correlated with telomere length in chronic stress (Cai et al., 2015b; Edwards et al., 2016).

There are several explanations for the lack of difference in mtDNA copy number in G1 Holocaust survivors as compared to controls. First, any elevated mtDNA copy number due to the experience may have reversed with time (Cai et al., 2015b). In particular, if release of mtDNA from apoptotic cells rather than intra-cellular mtDNA increase can explain the increase in mtDNA copy number observed in chronic stress and MDD (Lindqvist et al., 2018), discontinuation of apoptotic reaction upon removal of stressful stimuli may lead to complete recovery of elevation in mtDNA copy number, and explain why it cannot be observed decades later. Second, persistent mtDNA copy number changes may be dependent on MDD or other disease states (Cai et al., 2015b). While we were able to perform this test using mtDNA copy number in G1 Holocaust survivors with PTSD only, it is possible PTSD does not have the same molecular signature as MDD, or we do not have enough statistical power to identify it at current sample sizes. Third, Holocaust survivors may consist of highly resilient individuals who were able to survive both prolonged physical and psychological trauma. It was previously shown that Holocaust survivors have higher life-expectancy as compared to those who did not go through the same experience, potentially due to selection during the Holocaust (Sagi-Schwartz et al., 2013), and a genetic basis to this resilience was proposed (Lindqvist et al., 2018; Konečná et al., 2019). Genetic factors contributing to this resilience may contribute to recovery of elevated mtDNA copy number and maintaining telomere length (Konečná et al., 2019), though it is unclear if the same factors confer protection against PTSD and other disorders. Fourth, we may not be observing the right tissue for lasting molecular changes in Holocaust survivors. While an increase of mtDNA due to chronic stress was shown in multiple tissues including saliva, blood and liver in animal models (Cai et al., 2015b), a post-mortem study on suicide completers showed opposite changes in mtDNA levels in blood and dorso-lateral prefrontal cortex (Otsuka et al., 2017). As such, a lack mtDNA copy number difference between Holocaust survivors and controls in blood cells with rapid turnovers does not exclude lasting changes in mtDNA copy number in other tissues that may have important consequences on health and disease. Finally, other factors may influence and confound differences in mtDNA copy number between groups in our sample, including disease, epigenetic factors and ageing. While we were able to account for some of them using blood cell counts as proxy, an exhaustive assessment of these other factors are needed to fully account for their effects on mtDNA copy number.

Interestingly, we found that Holocaust experience of G1 Holocaust survivors was associated with lower mtDNA copy number in their children who also participated in this study. This cannot be completely accounted for by current mtDNA copy number of the G1 Holocaust survivors, and may be mediated through other biological or environmental factors that may be specific to descendants of Holocaust survivors. However, we found no replication of the effect of parental Holocaust experience on children’s mtDNA copy number in our whole cohort, where many participants did not indicate direct familial relationships with other participants. Whether this can conclusively dismiss the effect of parental Holocaust experience (or other traumatic experiences) in one’s mtDNA levels needs to be further investigated, ideally using a study design where all members of the family participate. This may no longer be possible with Holocaust survivors, many of whom have already passed away.

Finally, we observed higher rates of PTSD in G2 Holocaust survivor descendants than controls, consistent with previous findings (Yehuda et al., 1998), but not in G3, suggesting single generational inheritance of certain risk factors for PTSD, though we have no evidence mtDNA copy number elevation is one of them. While our results do not show evidence of maternal stress or PTSD effects on mtDNA copy number in their descendants, they do not rule out changes in mtDNA replication dynamics in oocyte development due to prenatal and gestational stress, and only targeted analysis on the relevant tissues may answer this question.

In summary, we did not find conclusive evidence of persistent mtDNA copy number changes or differences in age-related dynamics and inheritance of mtDNA in Holocaust survivors as compared to controls, and mtDNA copy number cannot be used as a marker for PTSD in Holocaust survivors or explain the inheritance of risk for PTSD in their descendants. This study is, to the best of our knowledge, the first comprehensive study of effect of stress and PTSD on mtDNA copy number dynamics and inheritance among Holocaust survivors and their descendants.

## Methods

### Participants in This Study

The study was conducted at Central European Institute of Technology (CEITEC), Masaryk University in Brno, Czech Republic. A part of DNA samples was obtained with the cooperation of the National Institute of Mental Health, Klecany, Czech Republic. All of the volunteers were Czechs or Slovaks (people with a similar geopolitical background). Participants had no brain trauma injury history or cognitive or mental impairment. While all Holocaust survivors and their descendants were fully or partially of Jewish origin, none of the controls were due to lack of Jewish persons without the Holocaust history in Czech Republic.

### Informed Consent and Ethical Approval

All participants were recruited through voluntarily responding positively to a public appeal presented by Masaryk University and Czech national media (Konečná et al., 2019), with the cooperation of the Jewish community of Brno and Prague. Written informed consent was obtained from all participants, except for in the cases of participants below the age of 16, where written informed consent was obtained from the next of kin/legal guardian. The study was conducted in accordance with the Declaration of Helsinki, and the protocol was approved by the Ethics Committee of Masaryk University, Brno, Czech Republic (project NV18-04-00559).

### Experience of Chronic Trauma in the Participants

G1 Holocaust survivors suffered from the extreme and chronic stress during the World War II, inclusive and often a combination of long-term humiliation, deprivation of basic human rights, life threating internment in prisons, death camps, hiding, false identities, fighting as partisans and assassination of family members. For participants in G2 and G3 generations, we obtained their relationships to G1 Holocaust survivors. For the participants in the control group, none were exposed to similar extreme and chronic stress.

### Evaluation of Post-Traumatic Stress Disorder (PTSD)

Markers of PTSD were evaluated using the 17-item civilian version of the PCL questionnaire (PCL-C), a general civilian version that is not linked to a specific event but to “a stressful experience from the past”, rather than PCL-M which is used in the case of military experiences and PCL-S for specific stressful events. Each item is rated 1 to 5 indicating the degree to which a participant has been affected by the item in the past month; a cut-off score of 30 was used to indicate a diagnosis of probable PTSD. The same questionnaire was administered to all participants by trained professionals.

### Isolation of Peripheral Blood Mononuclear Cells (PBMC) and DNA

A blood sample were collected from every participant in the years 2016 to 2019. Full blood count was performed on 136 participants using this blood sample. Peripheral blood mononuclear cells (PBMC) were isolated from whole blood samples using ficoll (Histopaque, Sigma) density gradient centrifugation. Genomic DNA was purified from PBMC samples by proteinase K (Roth) treatment, chloroform extraction, and isopropanol precipitation, as previously described. Quality and concentration of DNA were analyzed by agarose electrophoresis and spectrophotometrically using Thermo Fisher Scientific Nanodrop 2000.

### Quantification of mtDNA Copy Number Using Quantitative Polymerase Chain Reaction (qPCR)

qPCR was carried out using the TaqMan^®^ Universal PCR Master Mix, No AmpErase^®^ UNG. A nuclear genomic fragment was amplified from the RNase P gene using the TaqMan^®^ RNase P Detection Reagents Kit, and a fragment of the mitochondrial genome (positions 14747-15887) was amplified from the Cytochrome B (CYB) gene using TaqMan^®^ Gene Expression Assays—Hs02596867_s1—MT-CYB. qPCR was performed under the following conditions: incubation at 50°C for 2 min, denaturation at 95°C for 10 min, followed by 40 cycles of 15 s at 95°C and 1 min at 60°C. qPCRs of were carried out on 96-well plates; all samples were randomized for the plate they were analyzed on and their positions on the plates, a reference DNA sample was analyzed in triplicates across all plates, and all samples were run in duplicates. Threshold quantitation cycle (CT) was obtained of each sample including the reference DNA samples (REF); we obtain the difference between the mean CT (ΔCT) at for both genes between each sample and REF to correct for plate effects, before obtaining the difference between the CT of the two genes (ΔΔCT) as an estimate of mtDNA copy number. PCR runs were discarded if they failed to meet the following criteria: no template control (NTC) with a quantitation cycle (CT) > 38 cycles, sample with a CT <30 cycles.

### Quantification of mtDNA Copy Number From Whole-Genome Sequencing in 1000G Samples

We extracted reads mapping to the rCRS mitochondrial reference genome (NC_012920) from WGS in 2,558 individuals in Phase 3 of the 1000 Genomes Project (1000G). Reads mapping to chr20 and mtDNA with the following SAM flags are removed with –F 3852 using samtools (Li et al., 2009) to ensure unique and high quality mapping to chr20 and mtDNA reference genomes respectively: read unmapped (0 × 4), mate unmapped (0 × 8), not primary alignment (0 × 100), read fails platform/vendor quality checks (0 × 200), read is PCG or optical duplicate (0 × 400), and supplementary alignment (0 × 800). We quantified total read depth across all positions across chr20 and mtDNA and obtained mean coverage for each, and obtained a mtDNA copy number by correcting mean coverage over mtDNA with mean coverage over chr20. We then quantile normalized the measure to obtain a normalized mtDNA copy number.

### Haplogroup Calling Using mtDNA Variants Obtained From WGS of 1000G Individuals

We called mtDNA variants from WGS in 2,558 individuals in Phase 3 of the 1000 Genomes Project (1000G) using GATK v4 (McKenna et al., 2010; DePristo et al., 2011), obtaining 3,779 high quality biallelic SNPs. We use all 3,779 SNPs for assigning Haplogroups to each individual in 1000G using Haplogrep v2 (Weissensteiner et al., 2016). We built a genetic related matrix of all 1000G samples using all 3,779 mtDNA SNPs using LDAK (Speed et al., 2017) with options –ignore-weights YES –power -1 –hwe-stand NO, such that each mtDNA SNP contribute the same to the genetic covariance between individuals regardless of their minor allele frequencies, and are not standardized according to Hardy–Weinberg Equilibrium as if they were diploid. We then performed principal component analysis (PCA). As 626 SNPs are private to the 657 individuals with Haplogroup L, and within-Haplogroup diversity in Haplogroup L is greater than diversity across all other Haplogroups combined, we performed PCA on 3,153 mtDNA SNPs in the remaining 1,903 individuals to obtain PCs that show clustering of individuals by their mtDNA Haplogroups.

## Data Availability Statement

Datasets from 1000 Genomes Project Phase 3 (ENA Study Accession: PRJNA262923) were analyzed in this study. All other data is available on request to the authors.

## Ethics Statement

Written informed consent was obtained from all participants, except for in the cases of participants below the age of 16, where written informed consent was obtained from the next of kin/legal guardian. The study was conducted in accordance with the Declaration of Helsinki, and the protocol was approved by the Ethics Committee of Masaryk University, Brno, Czech Republic (project NV18-04-00559).

## Author Contributions

NC, MiF, JF, and IR designed the study. NC, SW, KK, and EC performed the experiments. NC performed the analysis. IR obtained funding from the Czech Health Research Council. MoF, KK, MiF, JF, MP, and IR and collected the data through public appeal presented by Masaryk University and Czech national media. NC interpreted the results and wrote the manuscript with the help of MiF, JF, NS, and IR with approval of all authors.

## Funding

This research was funded by the Czech Health Research Council, grant number NV18-04-00559, and by the project CEITEC 2020, grant number LQ1601, with financial support from the Ministry of Education, Youth and Sports of the Czech Republic under the National Sustainability Program II. NC is supported by the ESPOD Fellowship at the Wellcome Trust Sanger Institute and the European Bioinformatics Institute.

## Conflict of Interest

The authors declare that the research was conducted in the absence of any commercial or financial relationships that could be construed as a potential conflict of interest.
